# Factors Associated With Food Intake, Nutritional Status, and Function Among Nursing Home Residents With Dementia

**DOI:** 10.1093/geroni/igaa057.588

**Published:** 2020-12-16

**Authors:** Murad Taani, Adam Plotkin

**Affiliations:** 1 University of Wisconsin-Milwaukee, Milwaukee, Wisconsin, United States; 2 Cornell College, Cornell College, United States

## Abstract

Declined food intake is prevalent among long-term care (LTC) residents with dementia and associated with deleterious health outcomes. This study explores food intake, nutritional status, and function and its associated factors in LTC residents with dementia. Data from 82 LTC residents with dementia were used in this secondary analysis. In the primary study, appetite was assessed using the Short Nutritional Assessment Questionnaire (SNAQ). Dementia level, comorbidity, agitation, pain, mood, food intake, nutritional status, and function variables were extracted from the electronic medical record, including the most recent Minimum Data Set (version 3.0) assessment. The majority of residents were either malnourished or at risk of being malnourished and demonstrated a worse appetite than previously described in the literature. Comorbid illness, depressed mood, and appetite were associated with 37.1% of the variance in food intake over 30 days. Dementia level and appetite were associated with 22.2% of the variance in nutritional status. Food intake and nutritional status were associated with 29.1% of the variance in function. This study also highlights a new demographic that may require extra assistance in combating declined food intake: LTC residents with dementia who reside in a facility that follows restrictive food practices such as a kosher diet. The potential reversibility of factors associated with food intake and nutritional status provides opportunities for intervention.

